# Transcriptome-based biomarker gene screening and evaluation of the extracellular fatty acid-binding protein (Ex-FABP) on immune and angiogenesis-related genes in chicken erythrocytes of tibial dyschondroplasia

**DOI:** 10.1186/s12864-022-08494-9

**Published:** 2022-04-22

**Authors:** Ali Raza Jahejo, Sayyad Ali Raza Bukhari, Nasir Rajput, Nazeer Hussain Kalhoro, Imdad Hussain Leghari, Sayed Haidar Abbas Raza, Zhen Li, Wen-zhong Liu, Wen-xia Tian

**Affiliations:** 1 College of Animal Science, Shanxi Agricultural University, Jinzhong, 030801 China; 2 College of Veterinary Medicine, Shanxi Agricultural University, Jinzhong, 030801 China; 3 Department of Biotechnology, University of Sargodha, Sargodha, Pakistan; 4 Department of Poultry Husbandry, Sindh Agriculture University, Tandojam, Pakistan; 5 Sindh Institute of Animal Health, Karachi, Pakistan; 6 College of Animal Science and Technology, Northwest A&F University, Yangling, 712100 China

**Keywords:** Angiogenesis, Bone-deformity, Biomarker-genes, Broiler, Ex-FABP, Immunity

## Abstract

**Background:**

Tibial dyschondroplasia (TD) is a bone disorder in which dead chondrocytes accumulate as a result of apoptosis and non-vascularization in the tibial bone of broiler chickens. The pathogenicity of TD is under extensive research but is yet not fully understood. Several studies have linked it to apoptosis and non-vascularization in the tibial growth plate (GP). We conceived the idea to find the differentially expressed genes (DEGs) in chicken erythrocytes which vary in expression over time using a likelihood-ratio test (LRT). Thiram was used to induce TD in chickens, and then injected Ex-FABP protein at 0, 20, and 50 μg^.^kg^-1^ to evaluate its therapeutic effect on 30 screened immunity and angiogenesis-related genes using quantitative PCR (qPCR). The histopathology was also performed in TD chickens to explore the shape, circularity, arrangements of chondrocytes and blood vessels.

**Results:**

Clinical lameness was observed in TD chickens, which decreased with the injection of Ex-FABP. Histopathological findings support Ex-FABP as a therapeutic agent for the morphology and vascularization of affected chondrocytes in TD chickens. qPCR results of 10 immunity (*TLR*2, *TLR*3, *TLR*4, *TLR*5, *TLR*7, *TLR*15, *IL*-7, *MyD*88, *MHC*II, and *TRAF*6) and 20 angiogenesis-related genes (*ITGAV*, *ITGA*2, *ITGB*2, *ITGB*3, *ITGA*5, *IL1R*1, *TBXA2*R, *RPL*17, *F13A*1, *CLU*, *RAC*2, *RAP1*B, *GIT*1, *FYN*, *IQGAP*2, *PTCH*1, *NCOR*2, *VAV-like*, *PTPN*11, *MAML*3) regulated when Ex-FABP is injected to TD chickens.

**Conclusion:**

Immunity and angiogenesis-related genes can be responsible for apoptosis of chondrocytes and vascularization in tibial GP. Injection of Ex-FABP protein to thiram induced TD chickens decrease the chondrocytes damage and improves vascularization.

**Supplementary Information:**

The online version contains supplementary material available at 10.1186/s12864-022-08494-9.

## Background

Tibial dyschondroplasia (TD) is a leg bone deformity problem of broiler chicken that causes lameness. TD has a rising prevalence, and up to 30% of broiler flocks may be affected, and it is attributed to the death of chondrocytes due to low or no blood supply [1, 2]. Researchers are conducting in-depth research on TD to understand its pathogenicity. Although different aspects of TD occurrence have been well investigated since its report in 1965, its mechanism is still unclear [3]. Previously, TD was characterized by non-vascularized, un-mineralized, and non-viable cartilage in the tibial growth plate (GP), which failed to form bone [2, 4–6]. Therefore, any development regarding TD treatment is of utmost importance. It is very difficult to collect a large number of naturally occurring TD chickens for experimental purposes. Tetramethylthiuram disulfide (thiram) is a grain preservative often used in agriculture to induce artificial TD, often used to induce TD in chickens, which present the consistent results with natural TD [5, 7, 8]. Thiram affects the number of chondrocytes and destroys the normal proliferation, differentiation, and apoptosis of chondrocytes [1].

It has recently been reported that the extracellular fatty acid-binding protein (Ex-FABP) might have a therapeutic role in TD chickens [3] and osteoarthritic cartilage [9], which is upregulated during inflammation and reduces apoptosis [10]. It usually presents in chicken serum and represents the first extracellular protein able to selectively bind and transport fatty acid in extracellular fluids and blood [9]. However, the molecular impacts and therapeutic potential of the Ex-FABP protein are yet to be evaluated in TD chickens. This research assessed the therapeutic efficacy of Ex-FABP on TD chickens using histopathology and gene expression analysis. This experiment focused on the immunomodulatory function of chicken erythrocytes – the most common cells in circulation – before and after treatment with Ex-FABP based on the previous observation that TD regulates the expression of toll-like receptors (TLRs) 2, 3, 4, 5, and 21, as well as many immunological genes, including type I interferons (*IFN*) and interleukin (IL)-8 and angiogenesis-related genes such as integrins in TD induced chicken erythrocytes [11–13]. Multiple studies have reported the involvement of immunity and angiogenesis-related genes in thiram-induced TD chickens [12, 14]. Time scale regulation of gene expression needs to be explored, therefore initiated this two-phase experimental design; the first phase was based on a transcriptome sequencing analysis designed to classify differentially expressed biomarker genes on days 2, 6, and 15 after thiram treatment. The second phase of this study evaluated the mRNA expression of immunity and angiogenesis-related genes after the injection of Ex-FABP protein as a therapeutic intervention to thiram-induced TD chickens. Our findings will provide new evidence to understand the pathogenesis of TD and the therapeutic role of Ex-FABP in thiram-induced TD chicken.

## Results

### Differentially expressed genes (Phase 1)

**Fig. 1:**
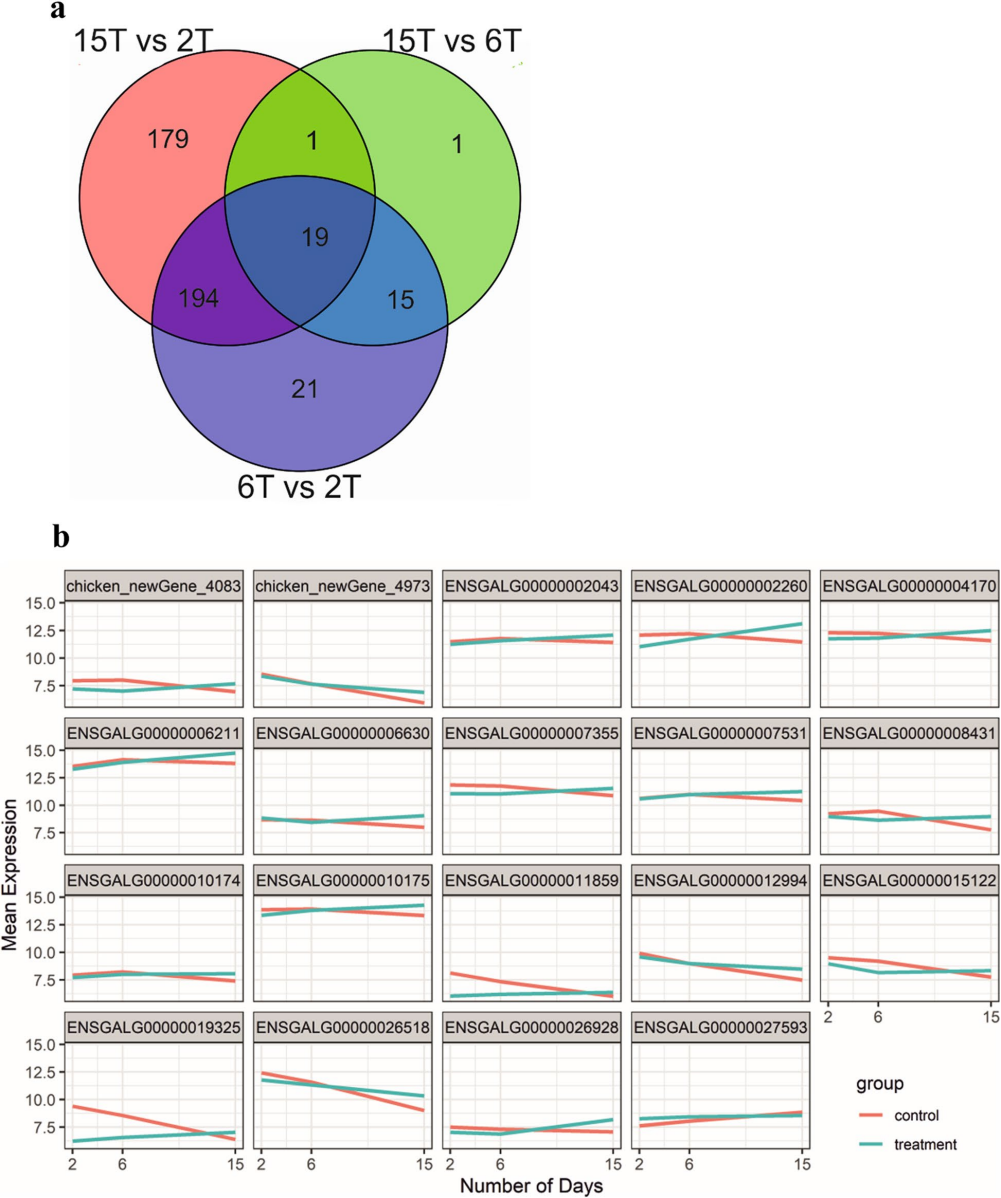
**a** Analysis of DEGs that show significance in the interaction of time and treatment, using Venn diagrams. There were 249, 393, and 36 DEGs identified on the 6th day vs 2nd day (6T vs 2T), 15th day vs 2nd day (15T vs 2T), 15th day vs 6th day (15T vs 6T). In total 19 DEGs commonly expressed in 6T vs 2T, 15T vs 2T, 15T vs 6T. **b** Graphical presentation of 19 common DEGs showing the mean of gene expression without time effect on 6th day vs 2nd day (6T vs 2T), 15th day vs 2nd day (15T vs 2T), 15th day vs 6th day (15T vs 6T). Red color represents the control group, and the blue color represents the thiram group

The analysis of differentially expressed genes (DEGs) between thiram induced TD broiler chickens (T) and untreated control broiler chickens (C) for days 2, 6, and 15 were analyzed using the likelihood-ratio test (LRT) modeling approach (Additional file 1). The LRT analysis was performed on all the raw counts (Additional file 2), to investigate the interaction of time and treatment. The genes that differ significantly in expression between control and treatment over different periods (days) were considered DEGs. The results revealed that a total of 678 genes were differentially expressed (*P* < 0.1). The number of DEGs on 2 day vs 6 day, 2 day vs 15 day and 6 day vs 15 day were 249 (101 up, 148 down), 393 (180 up, 213 down), and 36 (27 up, 09 down) respectively (Fig. 1a). The degree of expression change of these DEGs between the two samples is shown as a volcano plot in Supplementary Fig. 1.

There were 19 commonly DEGs in all the comparisons (Additional file 4), the expressed genes were WD Repeat Domain 33 (*WDR*33), Cytokine inducible SH2 containing protein (CISH), Adenosine deaminase (*ADA*), B-cell lymphoma 2 (*Bcl*-2) like 1 (*BCL2*L1), COMM domain containing 7 (*COMMD*7), Zinc finger FYVE-type containing 27 (*ZFYVE*27), SWI/SNF related, matrix associated, actin-dependent regulator of chromatin, subfamily a, member 1 (*SMARC*A1), Solute carrier family 35 member B2 (*SLC*35B2), Heat shock protein 90 alpha family class B member 1 (*HSP*90AB1), Eye-globin (*GBE*), ELKS/RAB6-interacting/CAST family member 1 (*ERC*1), TATA-box binding protein associated factor 4b (*TAF*4B), Uncharacterized LOC417973 (*LOC*417973), RUN domain containing 3A (*RUNDC*3A), Kruppel like factor 4 (*KLF*4), Uncharacterized protein (*VSIG*10L), Zinc finger protein 292 (*ZNF*292). Chicken_newGene_4083 and chicken_newGene_4973 lacks annotation in the database (Fig. 1b).

### Protein-protein interaction of 19 common DEGs

Based on the STRING database, protein-protein interactions (PPIs) of 19 common biomarker genes were predicted (Supplementary Fig. 2). The network consists of high to low combined scores. After analysis, 2 nodes and 8 protein pairs were obtained with a medium confidence level (0.150). BCL2L1 and CISH had the highest total score, 0.253. The second highest pair of BCL2L1 and KLF4 had a score of 0.184. The third composite score of the BCL2L1 and HSP90AB1 pair was 0.302. However, the lowest pair (TAF4B and WDR33) had a combined score of 0.427 (Additional file 6). The result demonstrated that BCL2L1 was the most important amongst the 19 common DEGs, which could be responsible for causing apoptosis and non-vascularization in the thiram-induced tibial GP.

### Gene ontology annotations

**Fig. 2:**
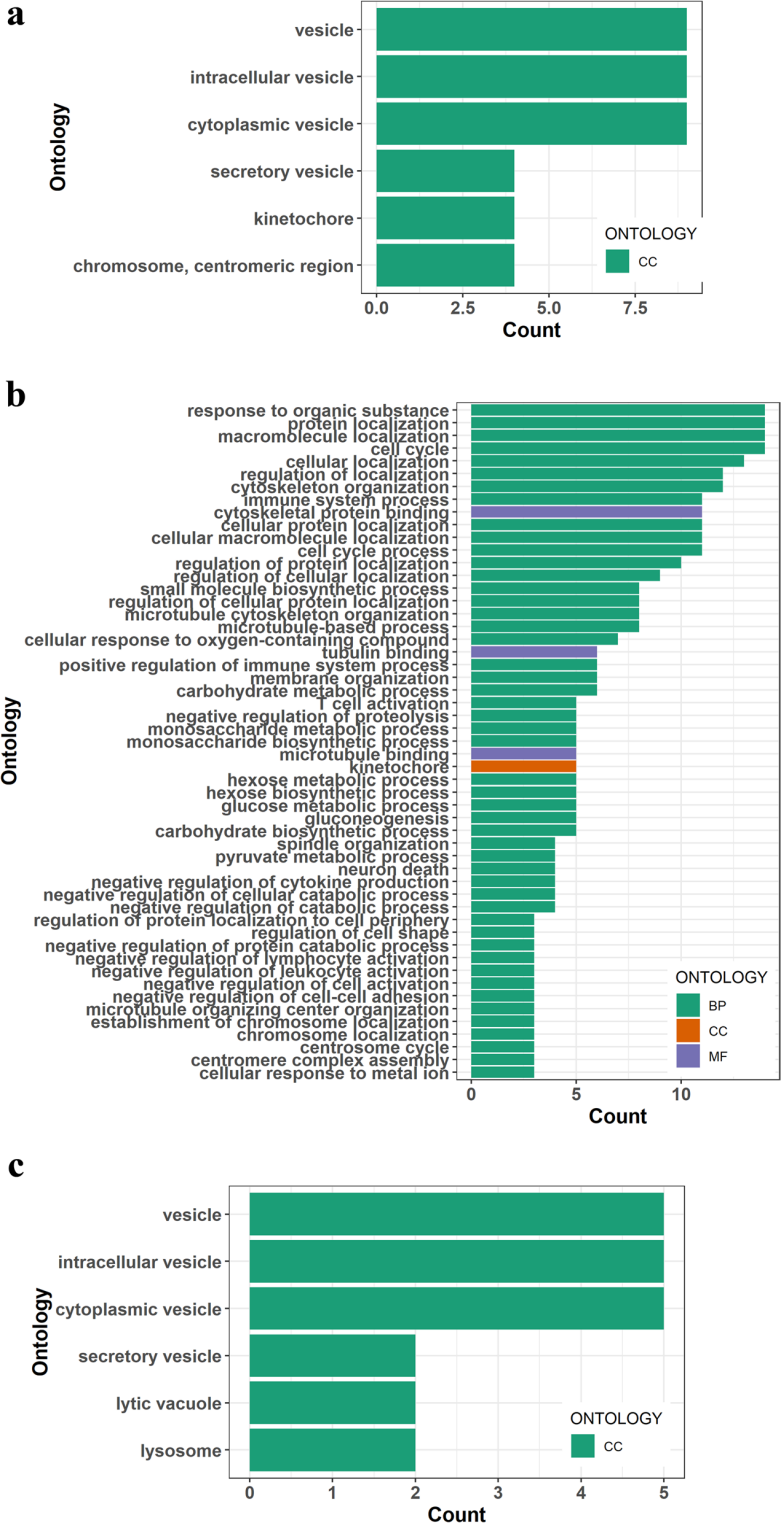
Gene ontology annotation of differentially expressed genes. **a** Up-regulated and down-regulated DEGs on 6th day vs 2nd day. **b** Up-regulated and down-regulated DEGs on 15th day vs 2nd day. **c** Up-regulated and down-regulated DEGs on 15th day vs 6th day. BP = biological process, CC = cellular process, MF = molecular functions. Counts = number of annoted genes

The gene ontology (GO) groups were allocated to a total of 678 DEGs (Additional file 7). Cytoplasmic vesicles (9 DEGs), intracellular vesicles (9 DEGs), and vesicles (9 DEGs) were the three enrichment groups of GO for 2 day vs 6 day and 6 day vs 15 day (Fig. 2a). The three enrichment categories of GO for 2 day vs 15 day were protein localization (14 DEGs), cell cycle (14 DEGs), cytoskeleton organization (14 DEGs) (Fig. 2b). The significance of genes related to cytoplasmic vesicle, intracellular vesicle, and vesicle in 2 day vs 6 day and 6 day vs 15 day reveal extreme fluctuations across different days (Fig. 2c). Moreover, the plot of DEGs in GO can be found with the *P* value ranked in Supplementary Fig. 3.

### Analysis of potential KEGG pathways

**Fig. 3:**
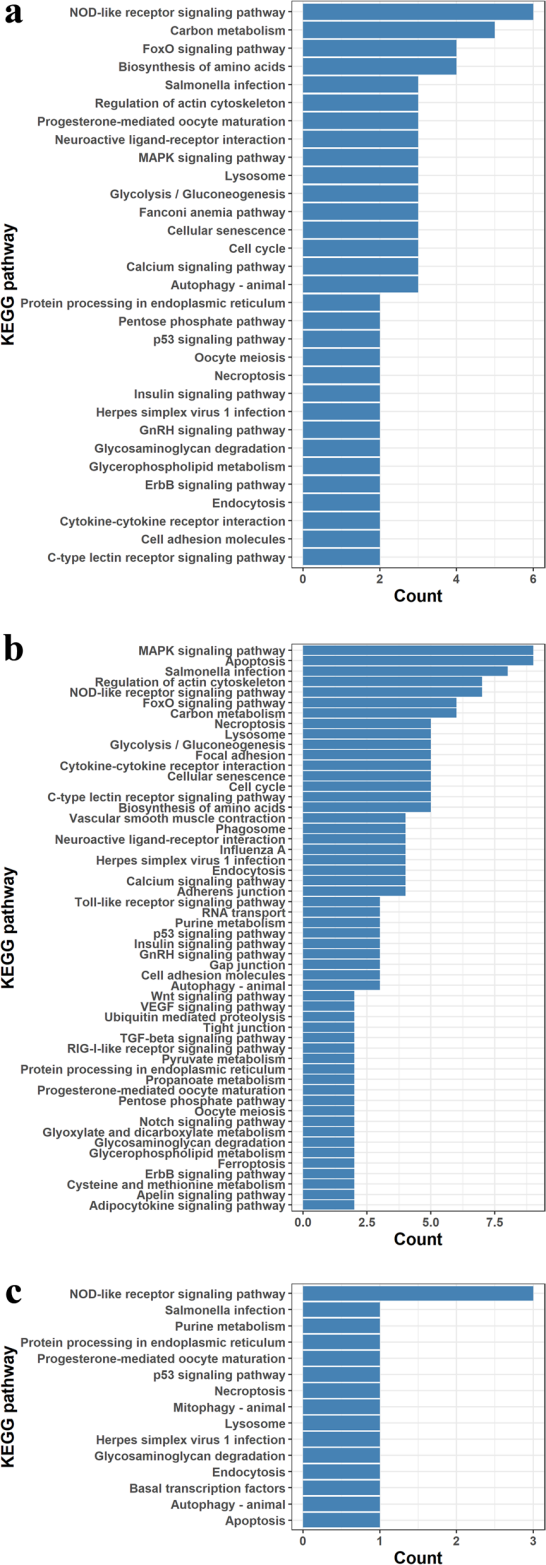
KEGG annotation of differentially expressed genes. **a** KEGG annotation of DEGs up-regulated and down-regulated DEGs on 6th day vs 2nd day. **b** KEGG annotation of DEGs up-regulated and down-regulated DEGs on 15th day vs 2nd day. **c** KEGG annotation of DEGs up-regulated and down-regulated DEGs on 15th day vs 6th day

The DEGs were annotated in different KEGG pathways (Additional file 9). The potential KEGG pathways for 2 day vs 6 day were the NOD-like receptor signaling pathway (6 genes) and biosynthesis of amino acids (4 genes) (Fig. 3a). The potential KEGG pathways of DEGs for 2 day vs 15 day were apoptosis (9 genes) and NOD-like receptor signaling pathway (7 genes) (Fig. 3b). The potential KEGG pathways of DEGs for 6 day vs 15 day were the NOD-like receptor signaling pathway (3 genes) and Glycosaminoglycan degradation (1 gene) (Fig. 3c). Moreover, the plot of KEGG pathways can be found with *P*value ranked in Supplementary Fig. 4.

### Clinical observation and posture of chicken (Phase II)

**Fig. 4:**
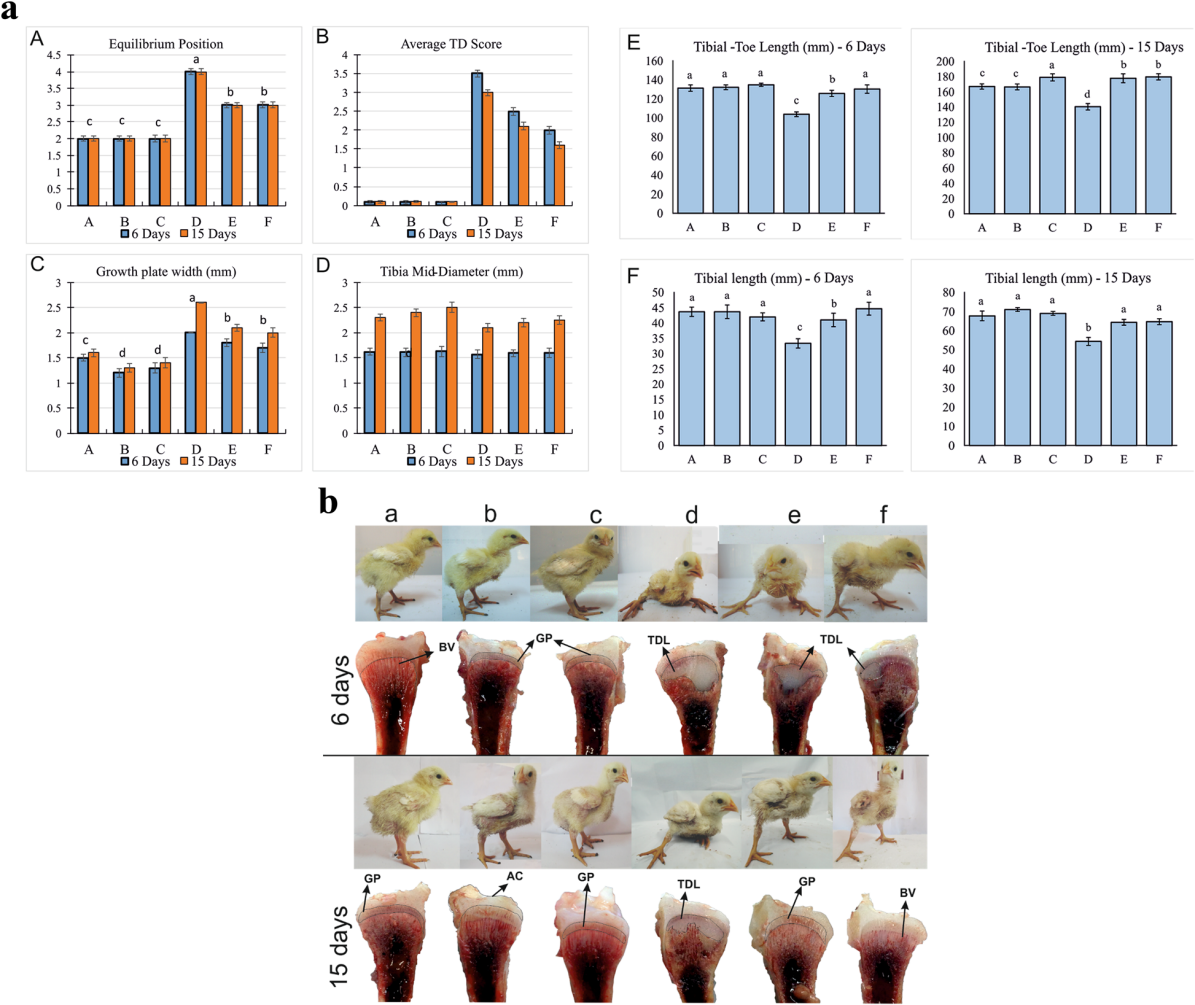
**a** Estimation of the equilibrium condition and the tibia parameters in the thiram induced and Ex-FABP supplementing broiler chicken on days 6 and 15. Ex-FABP was injected in group A, B, and C (0, 20, 50 μg^·^kg^−1^ respectively), D, E, and F (0, 20, 50 μg^·^kg^−1^ respectively). Whereas groups D, E, and F gave a diet containing 100 mg^.^kg^−1^ thiram. ^a–f^ indicates significant differences (*P* < 0.05). (**A**) Estimation of the equilibrium condition, (**B**) tibial dyschondroplasia occurrence score, (**C**) measurement of growth plate width, (**D**) measurement of growth plate midline-diameter, (**E**) measurement of the tibia to toe length, (**F**) measurement of tibia length. **b** The clinical and pathological changes in the thiram induced and Ex-FABP supplementing broiler chicken groups of days 6 and 15. Ex-FABP was injected in group A, B, and C (0, 20, 50 μg^·^kg^−1^ respectively), D, E, and F (0, 20, 50 μg^·^kg^−1^ respectively). Whereas groups D, E, and F were given a diet containing 100 mg^.^kg^−1^ thiram. BV, blood vessels; GP, growth plate; TDL, tibial dyschondroplasia lesions; AC, articular cartilage

The control group (A) of broiler chickens remained healthy and active throughout the experimental period without showing any signs of lameness. Group B and C, injected Ex-FABP and without thiram induction, show significantly good results. Thiram-induced TD broiler chicken group (D) showed several signs of depression, lameness, and failure to stand normally. Whereas broiler chickens in groups E and F, injected with Ex-FABP protein after thiram induction, expressed signs of recovery and significant improvements over group D, such as standing and walking. All the groups were evaluated on equilibrium condition (EC) scores for 6 and 15 days (Supplementary Fig. 5). The results suggest that the EC score was also significantly (*P* < 0.05) highest in group D for days 6 and 15 compared to other groups. However, the EC score was also significantly (*P* < 0.05) lower in groups E and F as compared to that of groups D and A (Fig. 4a-A).

### Pathology and morphometry of tibial bone

The tibial bone of thiram-induced TD broiler chickens was severely affected on days 6, and 15 in group D. However, the broiler chickens injected with Ex-FABP protein (E and F) expressed signs of recovery and showed less tibial lesions and decreased lameness as compared to that of group D (Fig. 4b). The pathological results supported the posture position (equilibrium condition) and tibial bone morphometry results.

The average TD score (Fig. 4a-B), growth plate width (Fig. 4a-C), tibia mid-diameter (Fig. 4a-D), tibia-toe-length (Fig. 4a-E), and tibia length (Fig. 4a-F) was severely affected in thiram induced TD group as compared to that of the control group. The broiler chickens in groups E and F showed improvement in all morphometric measurements.

### Histology and histomorphology of tibial bone

**Fig. 5:**
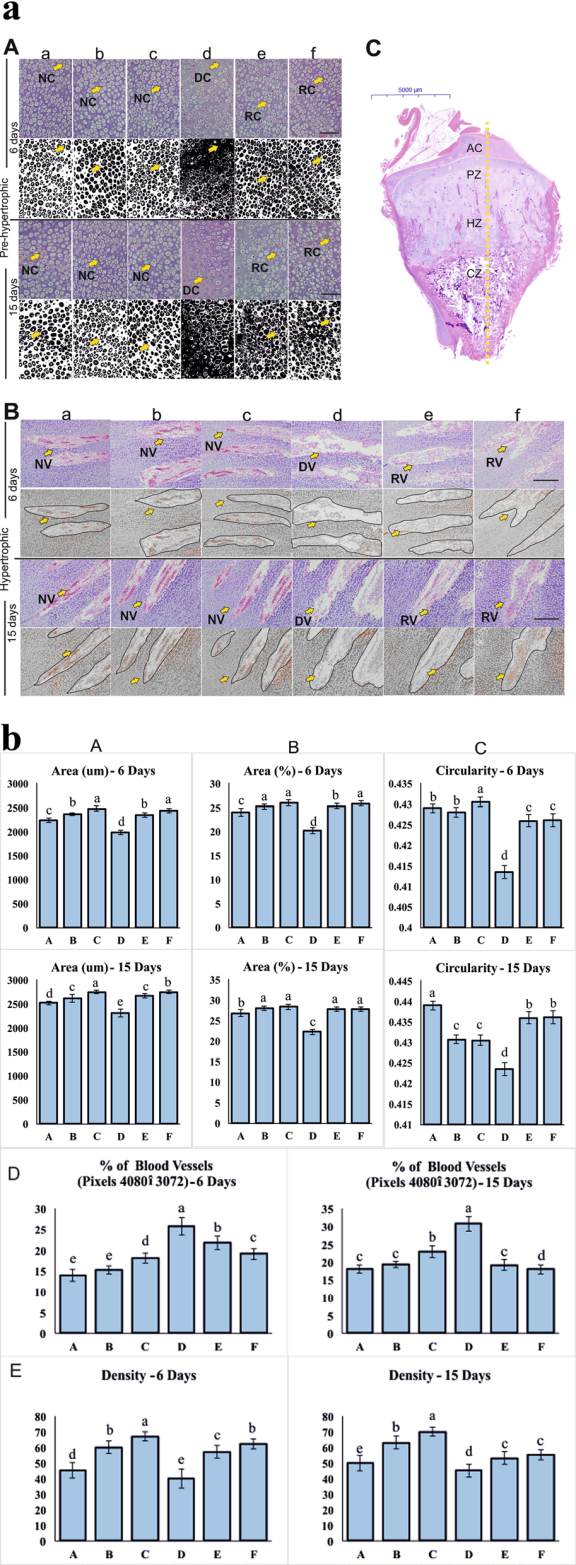
**a** Growth plate histomorphological changes of chondrocytes and blood vessels in the tibia (longitudinal section) for the thiram induced and Ex-FABP supplementing broiler chicken groups of days 6 and 15. Ex-FABP was injected in groups A, B, and C (0, 20, 50 μg^·^kg^−1^ respectively). D, E, and F (0, 20, 50 μg^·^kg^−1^ respectively). Whereas groups D, E, and F were given a diet containing 100 mg^.^kg^−1^ thiram. (**A**) H&E staining photographs of chondrocytes (Pre-hypertrophic zone) for days 6 and 15. NC, normal chondrocytes; DC, destroyed chondrocytes; RC, recovered chondrocytes. (**B**) H&E staining photographs of blood vessels (Hypertrophic zone) for days 6 and 15. NV, normal blood vessels; DV, destroyed blood vessels; RV, recovered blood vessels. (**C**) Zone-wise distribution of growth plate. AC, articular cartilage; PZ, proliferative zone; HZ, hypertrophic zone; CZ, calcification zone. **b** The histomorphological based quantification of chondrocytes and blood vessels for the thiram induced and Ex-FABP supplementing broiler chicken groups of days 6 and 15 using ImageJ software. Ex-FABP was injected in groups A, B, and C (0, 20, 50 μg^·^kg^−1^ respectively). D, E, and F (0, 20, 50 μg^·^kg^−1^ respectively). Whereas groups D, E, and F gave a diet containing 100 mg^.^kg^−1^ thiram. ^a–f^ indicates significant differences (*P* < 0.05). (**A**) Measurement of chondrocytes area (area=μm). (**B**) Measurement of chondrocytes area in percentage. (**C**) Measurement of chondrocytes circularity (area=μm). (**D**) The relative area of blood vessels. (**E**) The density of blood vessels

The histological examination of the tibial GP (Fig. 5a-C) revealed pyknosis and shortened nucleus in chondrocytes, and its pathological analysis showed the nucleus area of chondrocytes at days 6 and 15 in the thiram induced TD chicken group. However, the size and shape of chondrocytes and vascularization were normal in group A. The proliferation of chondrocytes was also affected mainly because of changes in size and shape. Moreover, the arrangements of chondrocytes became disordered in the pre-hypertrophic zone of thiram induced TD group, however, it recovered in Ex-FABP injection groups at days 6 and 15 (Fig. 5a-A). Similarly, angiogenesis also recovered in Ex-FABP injected groups (Fig. 5a-B).

The histomorphology of the microscopic figures was quantified with the ImageJ software, and the results of measurement of chondrocytes area (Fig. 5b-A), measurement of chondrocytes area in percentage (Fig. 5b-B), measurement of chondrocytes circularity (Fig. 5b-C), the relative area of blood vessels (Fig. 5b-D) and the density of blood vessels (Fig. 5b-E) suggests that thiram decreases the tibial bone vascularization and inhibits the growth of chondrocytes. The death of chondrocytes and changes in their structure were seen in thiram-induced TD group D. Moreover, the area, the density of blood vessels, and the circularity of chondrocytes improved in group E and F as compared to group D. We also observed that the blood vessels and chondrocytes began to recover in the Ex-FABP injection group on days 6 and 15 (Fig. 5b).

### Immunity and angiogenesis-related genes confirmation using PCR

**Fig. 6:**
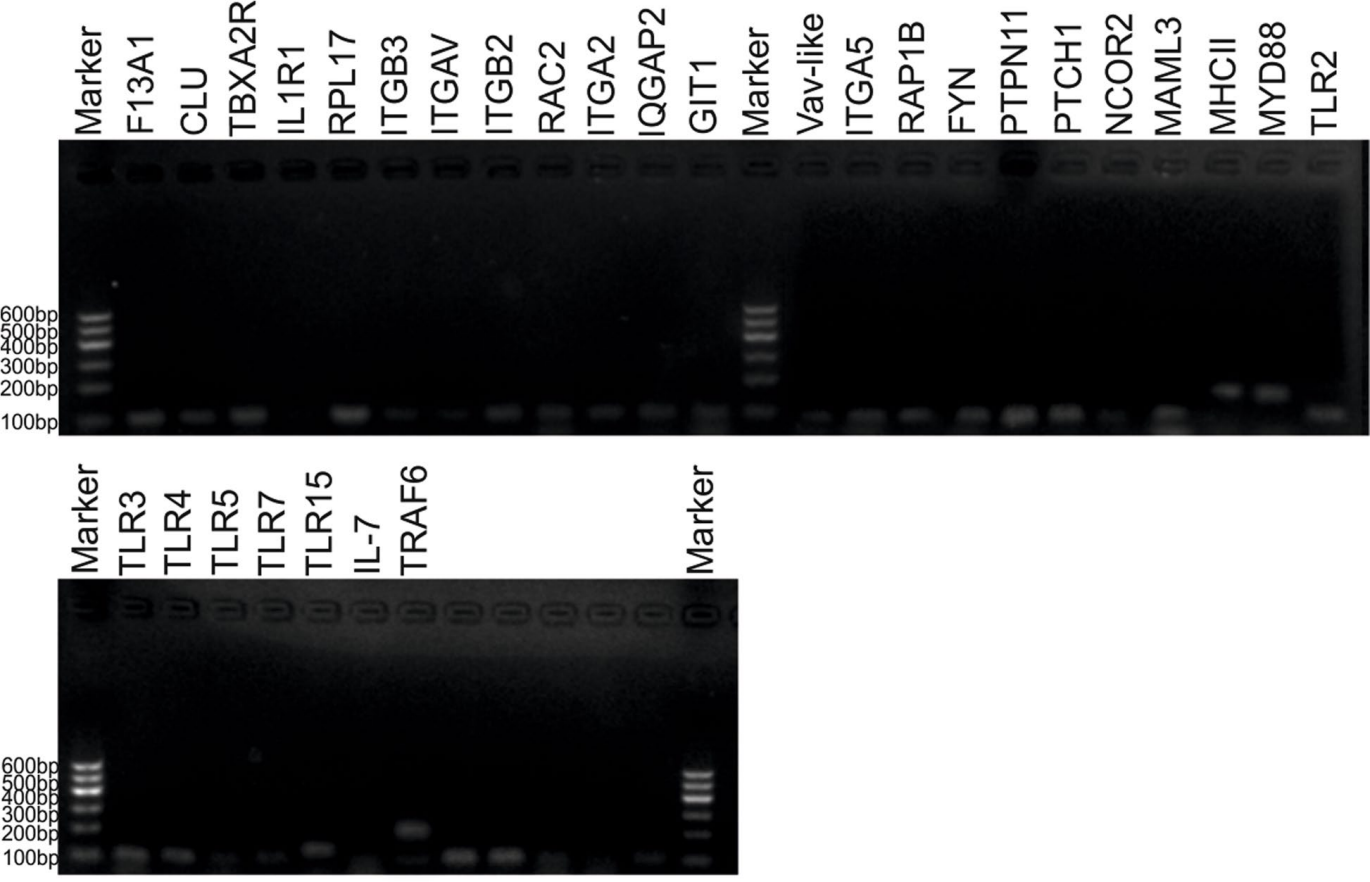
Expression pattern of angiogenesis and immune-related 30 genes using gel electrophoresis

The identification of immune and angiogenesis-related genes in the chicken erythrocytes was carried out by PCR (Fig. 6). All the immunity-related genes (PCR products) fall in between the base pair 100-200bp, including toll-like receptors 2, 3, 4, 5, 7, and 15 (*TLR*2, *TLR*3, *TLR*4, *TLR*5, *TLR*7, *TLR*15), interleukin 7 (*IL-7*), tumor necrosis factor receptor (*TNFR*) related factor 6 (*TRAF*6), major histocompatibility complex II (*MHC*II), myeloid differentiation primary response 88 (*MyD*88).

All the angiogenesis-related genes (PCR products) fall in between the base pair 100-300bp, including integrin α-v precursor (*ITGAV*), integrin β-2 precursor (*ITGB*2), integrin subunit beta 2 (*ITGB*2), integrin β-3 precursor (*ITGB*3), integrin α-IIb-like (*ITGA*5), interleukin-1 receptor type 1 precursor (*IL1R*1), thromboxane A2 receptor (*TBXA*2R), ribosomal protein L17 (*RPL*17), coagulation factor XIII A chain protein (*F13*A1), clusterin precursor (*CLU*), ras-related C3 botulinum toxin substrate 2 (*RAC*2), ras-related protein Rap-1b precursor (*RAP*1B), ARF GTPase activating protein (*GIT*1), tyrosine-protein kinase Fyn-like (*FYN*), IQ motif containing GTPase activating protein 2 (*IQGAP*2), protein repair homolog 1 (*PTCH*1), nuclear receptor co-inhibitor 2 (*NCOR*2), proto-oncogene vav (*VAV-like*), tyrosine-protein phosphatase Non-receptor type 11 (*PTPN*11) and mastermind-like protein 3 (*MAML*3). Moreover, the original, full-length version of gel electrophoresis images can be found in Supplementary Fig. 6.

### Protein-protein interaction of immunity and angiogenesis-related genes

PPIs of 30 immunity and angiogenesis-related genes were constructed and evaluated to predict their interactions. The network consists of high-low combined scores. A total of 2 nodes and 50 protein pairs obtained a low-confidence interaction score (0.150). MYD88 and TRAF6 had the highest combined score, 0.999. The second-highest combined score of TRAF6 and TLR4 was 0.997. ITGAV and ITGB3 had the third-highest overall score of 0.996 (Additional file 13).

In contrast, the last pair (FYN and LOC419429) had the lowest overall score of 0.41 (Supplementary Fig. 7). These results suggested the dominance of immunity-related genes when angiogenesis and immunity-related genes interact, as reflected by their best-combined score. It can be speculated that immunity-related genes are more important than angiogenesis-related genes, dependent on the immunity-related genes.

### qPCR of immune and angiogenesis-related genes

We observed the effect of Ex-FABP on the expression of immunity and angiogenesis-related genes, specifically at days 6 and 15 using qPCR.

**Fig. 7:**
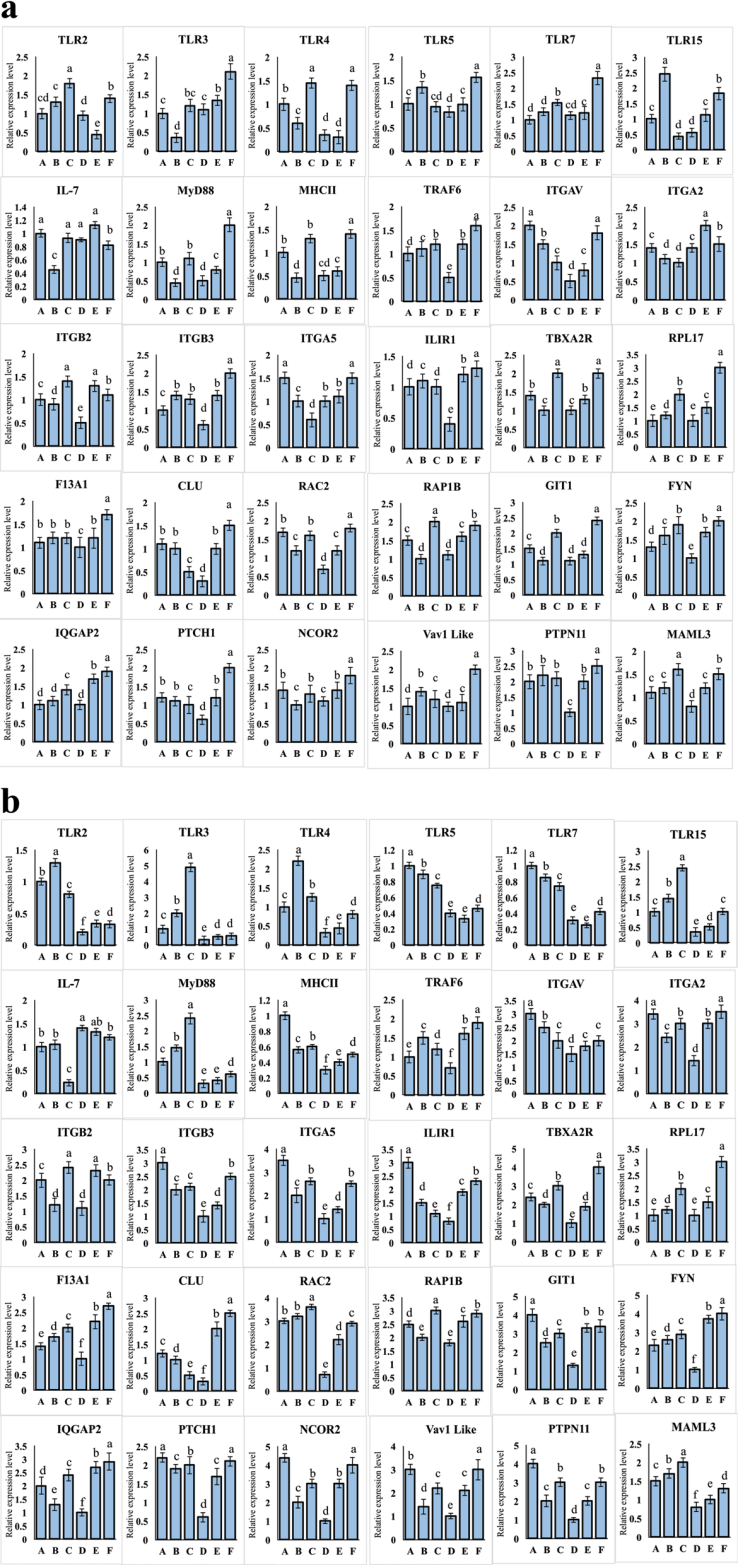
**a** Expression changes of chicken angiogenesis and immune-related 30 genes in the thiram induced and Ex-FABP supplementing broiler chicken groups of day 6. Ex-FABP was injected in groups A, B, and C (0, 20, 50 μg^·^kg^−1^ respectively). D, E, and F (0, 20, 50 μg^·^kg^−1^ respectively). Whereas groups D, E, and F were given a diet containing 100 mg^.^kg^−1^ thiram. ^a–f^ indicates significant differences (*P* < 0.05). **b** Expression changes of chicken angiogenesis and immune-related 30 genes in the thiram induced and Ex-FABP supplementing broiler chicken groups of day 15. Ex-FABP was injected in groups A, B, and C (0, 20, 50 μg^·^kg^−1^ respectively). D, E, and F (0, 20, 50 μg^·^kg^−1^ respectively). Whereas groups D, E, and F were given a diet containing 100 mg^.^kg^−1^ thiram. ^a–f^ indicates significant differences (*P* < 0.05)

On day 6, the immunity-related genes *TLR*2, *TLR*4, *TLR*5, *TLR*7, *TLR*15, *MyD*88, *MHC*II, *TRAF*6 were significantly downregulated (*P* > 0.05) in group D as compared to control group A. Whereas, and the same genes were significantly upregulated (*P* < 0.05) in the 50 mg^.^kg^-1^ Ex-FABP injected group F as compared to both groups D and A. The immunity-related gene *IL*-7 was significantly (*P* < 0.05) upregulated in group D while downregulated when supplemented with the 50 mg^.^kg^-1^ Ex-FABP protein as in group F. The higher expression of *IL*-7 results suggested that the healing process is activated after the toxic effect of thiram. The process got reversed when Ex-FABP was injected. The angiogenesis-related genes *ITGAV*, *ITGB*2, *ITGB*3, *ITGA*5, *ILIR*1, *TBXA2*R, *F13A*1, *CLU*, *RAC*2, *RAP1*B, *GIT*1, *FYN*, *PTCH1*, *NCOR*2, *PTPN*11, and *MAML*3 were significantly (*P* < 0.05) downregulated group D as compared to A. Moreover, the same genes were significantly (*P* < 0.05) upregulated in group F (Fig. 7a).

On day 15, TLR3 was also downregulated (*P* > 0.05) in addition to the previously described immunity-related genes in group D as compared to group A. Similar to what was observed at day 6, all of these genes were found to be significantly upregulated (*P* < 0.05) in the group F as compared to that of group D and group A. Same trend was found for *IL*-7 as well. The angiogenesis-related genes *ITGA*V, *ITGA*2, *ITGB*2, *ITGB*3, *ITGA*5, *ILIR*1, *F13A*1, *CLU*, *RAC*2, *RAP1*B, *GIT*1, *FYN*, *IQGA*P2, *PTCH*1, *NCOR*2, *Vav 1 like PTPN*11 and *MAML*3 were significantly (*P* < 0.05) downregulated in group D as compared to group A. Moreover, the same genes were significantly (*P* < 0.05) upregulated in group F (Fig. 7b).

## Discussion

TD is one of the major metabolic cartilage diseases in which the tibial GP cartilage fails to undergo osteogenic transition leading to the retention of thickened white opaque avascular cartilage plug [1, 15]. TD chickens were found with shrink cells, irregular chondrocyte columns, nuclear retraction, dissolution, and apoptosis [16]. Ex-FABP is reported to prevent vascularization and apoptosis therefore, we conceptualized its role in TD. We screened some biomarker genes based on transcriptome sequencing responsible for apoptosis and then selected immunity and angiogenesis-related genes to evaluate the effect of Ex-FABP in the erythrocytes of thiram-induced TD chickens.

Differential expression analysis was performed using LRT as implemented in DESeq2. LRT finds genes differentially expressed between a full model and a reduced model. Our full model was “time + treatment + time: treatment” where “time: treatment denotes interaction effect between time and treatment. Our reduced model was “time + treatment”. The rationale of such a design was to understand the genes affected by the interaction of time and treatment. Our model finds the genes of which expression alters significantly over different periods (days). The effect imparted by only time was controlled in the model to purely assess the genes due to the interaction of time and treatment and not the time or treatment alone. Using such a model, we found 19 common differentially expressed genes, which are *WDR*33, *CISH*, *ADA*, *BCL*2L1, *COMMD*7, *ZFYVE*27, *SMARCA*1, *SLC*35B2, *HSP*90AB1, *GBE*, *ERC*1, *TAF*4B, *LOC*417973, *RUNDC*3A, *KLF*4, *VSIG*10L, *ZNF*292.

Furthermore, we found that the most protein-protein interaction score belonged to *BCL*2L1 compared to other genes, which stated that BCL2L1 might play a major role in TD. Rath et al. [4] reported that the early effects of thiram on the GP might be the failure of genes encoding vascular endothelial growth factor (VEGF) receptors and Bcl-2 resulting from endothelial cell death, which compromise vascularization, cartilage remodeling, and the removal of dead chondrocytes leading to TD lesions. Similarly, we have also found that *BCL2*L1 might be responsible for the apoptosis of chondrocytes and vascularization in the GP. We speculated that these common genes could cause apoptosis, further studying in our ongoing experiments.

The second phase of this study was based on tibial bone histopathology. We evaluated the immunity and angiogenesis-related genes of chicken erythrocytes after the injection of Ex-FABP protein to thiram-induced TD chickens. Based on histopathological results, we found that thiram may cause erythrocytes to respond and to regulate the expression of immunity and angiogenesis-related genes resulting in apoptosis of chondrocytes. Previously, cartilage cell damage has been reported due to the apoptosis process and abnormal protein secretion in the cartilage cells [17, 18]. Moreover, thiram destroyed the regular columns of chondrocytes, decreasing its count, while Ex-FABP treatment enhanced that count [1, 19–21].

Moreover, the increase of fatty acids in the cellular microenvironment may increase the number of cells that undergo degradation and death during inflammation [22]. The binding of these fatty acids by Ex-FABP plays a key role in preventing chondrocyte apoptosis [10], and inhibiting the synthesis of Ex-FABP in different cell cultures can significantly reduce cell survival. Further reported that the apoptotic rate of chondrocytes and myogenic cells decreases when transfected with Ex-FABP cDNA, indicating that Ex-FABP can reduce apoptosis [10].

Our results demonstrate that broiler chicken erythrocytes can constitutively express 10 immune-related genes at the mRNA level, namely *TLR*2, *TLR*3, *TLR*4, *TLR*5, *TLR*7, *TLR*15, *IL*-7, *MyD*88, *MHC*II, and *TRAF*6. In addition, angiogenesis-related genes, namely *ITGA*V, *ITGA*2, *ITGB*2, *ITGB*3, *ITGA*5, *IL1R*1, *TBXA2*R, *RPL*17, *F13A*1, *CLU*, *RAC*2, *RAP*1B, *GIT*1, *FYN*, *IQGAP*2, *PTCH*1, *NCOR*2, *VAV-like*, *PTPN*11, and *MAML*3. Based on histopathology and mRNA results, it was speculated that Ex-FABP injection might regulate apoptosis and vascularization of tibial GP. To study the transcription of immune and angiogenesis-related genes in chicken erythrocytes of thiram-induced TD, we feed thiram to broilers at a concentration of 100 mg^.^kg^-1^ and found that chondrocytes and vascular endothelial cells in the TD broiler GP undergo apoptosis. It has been reported in several studies that TLRs can cause cell apoptosis when viruses, bacteria, and toxins are found in the organism [23–25]. The transcription results of mRNA indicated that Ex-FABP protein might play a key role in preventing apoptosis induced by thiram. However, the specific molecular mechanism is still not explained.

We found that the *MyD*88 gene was significantly upregulated on day 6 and downregulated on day 15 in group F. However, a study on the rat model evaluated that blocking *MyD*88 can reduce apoptosis [26]. It could be speculated that apoptosis reduced on day 15. Similarly, up-regulation of *TRAF*6 was also related to apoptosis [27], which we found upregulated on day 6 and 15 in group F. *MHC*II reported to cause apoptosis, was found downregulated on day 15 [28]. *TLR*7 that was downregulated on day 15 is also responsible for inducing apoptosis [29]. Similarly, activated *TLR*4 on day 6 was downregulated on day 15 in group F. The activation of the *TLR*4-*MyD*88 mediated by stress promotes apoptosis by angiotensin II [30]. Moreover, *TLR*5 also had similar results, downregulated on day 15. *TLR*5 may cause podocyte apoptosis [31]. Furthermore, *IL*-7 can promote the survival of T cells by inactivating the death promoter (Bad) associated with the pro-apoptotic protein B-cell lymphoma 2 [32]. In-addition, inflammatory factors (such as endotoxin and IL-6) can induce the synthesis of Ex-FABP. On the other hand, the reduction of Ex-FABP synthesist results in a lack of proliferation and differentiation of chondrocytes and, ultimately, apoptosis of chondrocytes [10].

Thiram can also cause membrane damage, inactivate bone morphology and angionecrosis [1, 33]. We found the expression of CLU to be very similar at both 6th and 15th days. It prevents apoptosis and necrosis caused by genotoxicity and oxidative stress [34]. Moreover, the *F13A1* protein coagulates blood when interacting with vascular endothelial growth factor receptor 2 (VEGFR2) and integrin αVβ3. It plays a vital role in angiogenesis and tissue repairing [35]. *F13A1* was found upregulated on day 6 and 15 in Ex-FABP supplementary groups, which suggest tissue repairing is in process. Recent studies reported that *TBXA2*R is involved in platelet aggregation and activation, thereby activating various signal transduction cascades to control various cellular processes, such as smooth muscle vasoconstriction, response to inflammation, cell adhesion, movement, proliferation, and cell survival. Reports have further suggested dramatic cell killing when the level of *TBXA2*R downregulated [36, 37]. *TBXA2*R was found upregulated in group F on days 6 and 15. Transfection of chondrocytes and myoblasts with antisense Ex-FABP cDNA can inhibit cell proliferation and induce cell apoptosis. In addition, inflammatory stimuli and pathological conditions significantly increase the expression of Ex-FABP [38]. *ILI*R1 is a cell surface receptor, a mediator of inflammation, and controls responses to injury or stress [39]. *ILI*R1 was found upregulated on day 6 and downregulated on day 15, suggesting that tibial dyschondroplasia due to thiram induction causes injury gets reduced on day 15. It is reported that the ribosomal pathway is related to cell growth, proliferation, and apoptosis, which mediates the angiogenesis of injury or stress [40]. If the expression of *RPL*17 is down-regulated, it will lead to VSMC proliferation; *RPL*17 has a tumor suppressor effect [41]. However, in this study, we found that the expression of RPL17 was upregulated on days 6 and 15. Ex-FABP is also called a survival protein, which prevents apoptosis in the chondrocytes [10]. *RAC*2 and *RAP*1B belong to the ras family, while *GIT*1 and *IQGAP*2 belong to the GTPase activating protein family. According to reports, the Ras family and GTPase activating protein family regulate angiogenesis [42]. However, we found that *RAC2* expression was significantly downregulated on day 6 and upregulated on day 15 in group F, suggesting that angiogenesis increased on day 15. This study evaluated integrin-related genes downregulated on the 6th and 15th days in thiram-treated chickens, which upregulated significantly (*P* < 0.05) when injecting 20 and 50 μg^.^kg^-1^ Ex-FABP. Our results are consistent with Mehmood et al. [5]. They studied the expression of *ITGB*3 in chickens treated with thiram and found the downregulation of the *ITGB*3 gene on the 14th day, which gets upregulated at the usage of ligustrazine.

Similarly, we found integrin genes, including *ITGB*3, regulated when injected Ex-FABP to thiram-induced TD chickens at day 15. Several studies have reported that integrins are involved in angiogenesis and are considered potential anti-angiogenic targets [43, 44]. High FABP5 expression enhances fatty acid transport to cancer cells used as a new energy source to meet the needs of rapid cell growth. In addition, excess fatty acids are transported into the nucleus, where they act as signal molecules to stimulate its nuclear receptor peroxisome proliferator-activated receptor gamma (PPAR gamma). PPAR gamma regulates genes that increase aggressiveness, resulting in decreased apoptosis and increased angiogenesis [45–48]. We observed a dramatic loss of cell viability and strong inhibition of cell proliferation and differentiation. However, when Ex-FABP was used to transfect chondrocytes, it could prevent cell apoptosis. It has also been reported by the study of Di Marco et al. [10], who suggest that Ex-FABP is a constitutive survival protein whose expression and activation are essential to protect cartilage cells from cell death.

## Conclusion

In conclusion, chicken erythrocytes significantly express immunity (*TLR*2, *TLR*3, *TLR*4, *TLR*5, *TLR*7, *TLR*15, *IL*-7, *MyD*88, *MHC*II, and *TRAF*6) and angiogenesis-related genes (*ITGA*V, *ITGA*2, *ITGB*2, *ITGB*3, *ITGA*5, *IL1R*1, *TBXA2*R, *RPL*17, *F13A*1, *CLU*, *RAC*2, *RAP*1B, *GIT*1, *FYN*, *IQGAP*2, *PTCH*1, *NCOR*2, *VAV-like*, *PTPN*11, and *MAML*3) which may cause apoptosis of chondrocyte and vascularization in the GP. However, the injection of Ex-FABP protein in thiram-induced TD chickens prevents apoptosis and regulates angiogenesis. The most important genes found in this study are integrin and TLRs that may change the morphology of chondrocytes in the growth plate. In addition, we found 19 biomarker genes, which may be related to the apoptosis of chondrocytes, such as the B-cell lymphoma 2 (*Bcl-2*) gene. These 19 (DEGs) genes will be studied in our ongoing experiments. It is recommended to inject 50 μg^.^kg^-1^ Ex-FABP protein on day 10 in broiler chickens to prevent TD. Our findings provide new evidence to understand the pathogenesis to prevent TD in the future.

## Materials and methods

### Experimental design

This study was divided into two phases.

The first phase was conducted to investigate the comparison between control and thiram-induced TD chickens of days 2, 6, and 15 based on transcriptome sequencing.

Broiler chickens were induced with TD in the second phase and divided into 6 and 15 days. The Ex-FABP protein was supplemented to TD chickens, followed by mRNA expression analysis of 30 biomarker genes related to angiogenesis and immunity. In addition, histomorphology of the tibia was performed.

### Induction of TD and blood collection (Phase-1)

In the first phase, a total of 24 broiler chickens (a day-old) (Shanxi Daxiang Farming Community, Shanxi, China) were obtained and supplied *ad-libitum* feed and water. On the seventh day, broiler chickens were fed for 48 hours with 100 mg^.^kg^-1^ thiram to induce TD, following the instruction given by Tian et al. [7]. After the TD was induced for 48 hours, the controls (C) and thiram (T) groups were randomly allocated to all 24 broiler chickens differentiated as day 2, day 6, and day 15. Blood extracts were obtained from the brachial vein until scarification on days 2, 6, and 15.

### RNA extraction, library construction, and RNA-Sequencing

The blood samples for the control and treatment groups were collected on days 2, 6, and 15. The total RNA was extracted from all samples using TRIzol reagent (Invitrogen, Carlsbad, CA, USA) as previously described by Niu et al. [49]. As per manufacturer protocol, the library construction and RNA-Seq were performed at Beijing Bio Marker Technologies (Beijing, China). In brief, the purity of RNA was analyzed using a Nano Photometer spectrophotometer (IMPLEN, CA, USA). The concentration of extracted RNA was measured using Qubit RNA Assay Kit. in a Qubit 2.0 Fluorometer (Life Technologies, CA, USA) was used to measure the RNA concentration. Subsequently, the integrity of the RNA was assessed using the RNA Nano 6000 Assay Kit (Agilent Technologies, CA, USA). A total amount of 1 mg RNA per sample was used as input material for the RNA sample preparations. NEBNext UltraTM RNA Library Prep Kit was used to synthesize sequencing libraries (NEB, USA) following the manufacturer's recommendations. NEBNext Poly (A) mRNA Magnetic Isolation Module (NEB, E7490) was used to isolate mRNA from the total RNA. The cDNA library was constructed following the manufacturer’s instructions of NEBNext Ultra RNA Library Prep Kit for Illumina (NEB, E7530) and NEBNext Multiplex Oligos for Illumina (NEB, E7500). In brief, the enriched mRNA was fragmented and used to synthesize the first-strand cDNA, followed by end-repair/dA-tail and adaptor ligation. Agencourt AMPure XP beads (Beckman Coulter, Inc.) were used to isolate suitable-sized fragments. Subsequently, the samples were treated with 3 μL USER Enzyme (NEB, USA) 37°C for 15 minutes, followed by 5 minutes at 95°C before PCR. Universal PCR primers and Index (X) Primer were used to preserve the fidelity of the synthesized cDNA Phusion High-Fidelity DNA polymerase for subsequent PCR. Finally, the PCR items (AMPure XP system) were filtered, and the Agilent Bioanalyzer 2100 system was tested for library consistency. Eventually, the cDNA libraries were sequenced using an Illumina HiSeq™ 2500 sequencing device on a flow cell.

### Transcriptome analysis using reference genome-based reads mapping

As a quality control step, adaptor sequences and low-quality sequences were cleaved using TrimGalore (version 0.6.6) (https://github.com/FelixKrueger/TrimGalore). The reads whose quality score was less than 20 in more than 20% of the bases were rejected. The clean reads were then mapped to the chicken genome (*gallus gallus 5.0*) using Tophat2 [50] in the bash command-line interface. Raw counts of gene expression were counted using HTSeq (0.13.5). The raw counts were imported in R for differential gene expression using DESeq2.

### Identification of differential gene expression

The DESeq2 (1.28.1) package was used for differential expression analysis in the R environment (4.0.3). Anders, Huber [51] provides a statistical model for determining the differential expression in the database using a model based on the negative binomial distribution. Benjamini and Hodgeberg was used to control the false discovery rate (FDR) to adjust the *P*value obtained. The likelihood ratio test (LRT) was used for the expression analysis. LRT finds differentially expressed genes between a full model and a reduced model. “Time + treatment + time: treatment" was our complete model, where "time: treatment” denotes the interaction of time and treatment. “Time + treatment" was our reduced model. This design was to understand the genes that are influenced by time and treatment. Our model finds genes that shift their expression dramatically over various periods. In the model, the effect imparted by just time or just treatment was controlled to purely test the genes that are due to the interaction of time and treatment and not time alone.

### Annotation of differentially expressed genes

The annotation of the DEGs was performed by gene set enrichment analysis as implemented in cluster Profiler (3.16.1) [52]. Gene ontology (GO) and Kyoto Encyclopedia of Genes and Genomes (KEGG) annotation was performed with an enrichment *P*-value of < 0.1 [53]. The resulting annotations were plotted using ggplot2 (3.3.2) [54] or wrapper functions of cluster Profiler.

### Protein-protein interaction (PPI) network construction

In order to find which gene or pair of the gene as protein interacts best within the 19 common DEGs, the STRING database was used for protein-protein interaction (PPI) following Mering et al. [55]. The STRING database is used to find the predicted PPI interaction based on the detail available in the database. This operating methodology for the database is based on the prediction process (neighborhood gene, gene fusion, co-occurrence, experiment co-expression, database, and text mining). In order to find their PPIs, we evaluated the typical 19 DEGs and used a combined score above > 0.9 as the cut-off point. The STRING database used a low confidence PPI (0150) (*Gallus gallus*).

### Induction of TD and blood collection (Phase-2)

In the second phase, 120 broiler chickens (a day-old) were obtained from (Shanxi Daxiang Farming Community, Shanxi, China). Firstly, chickens were reared on *ad-libitum* feed and water for seven days. Then, all the birds were randomly distributed into 6 groups — A, B, C, D, E, and F; each group contains 20 birds. Subsequently, A, B, and C were treated with 0, 20, and 50 μg^.^kg^-1^ Ex-FABP protein, respectively, through the injected method. Additionally, D, E, and F were allocated for TD-induction and fed with 100 mg^.^kg^-1^ thiram for 48 hours, as Tian et al. [7] recommended. Finally, TD-induced broiler chickens were injected with 0, 20, and 50 μg^.^kg^-1^ Ex-FABP protein for A, B, and C groups. After that, blood samples of 2.5 mL were taken from a brachial vein from each bird on days 6 and 15 before scarification.

### The posture of chicken and tibial bone morphometry

All the broiler chickens were routinely checked as previously described by Alves et al. [56]. Photographs were taken to evaluate the equilibrium condition (EC), as shown in Supplementary Fig. 3. The body posture abnormality was evaluated on the score based, the perpendicular line that was drawn in front of the footpad; score 1—leaned back and was highly erect, score 2—fell on the footpad area, score 3—prostrated equilibrium condition or a leaning forward posture/ unable to stand score 4—. Moreover, tibiae samples of 4 birds were taken at 6 and 15 post-day treatment. For morphological analysis, as described by Rath et al. [57], a Digital caliper (SATA91511, TATA Corporation, Shanghai, China) was prepared to measure the left sagittal section of the proximal tibial GP to measure the length and width of the tibia. Following Pines et al. [58] and Simsa et al. [59], the average TD score was calculated. Subsequently, the images were captured before and after the thiram treatment as per instructions are given by Rath et al. [57].

### Histology and its quantification

For histological analysis, the left sagittal sections of the tibia were fixed in 4% paraformaldehyde [57]. Routine paraffin sections and hematoxylin and eosin staining were performed as described by Tian et al. [7]. Subsequently, the relative area of the blood vessel (the area of the region of interest divided by the total area to determine the percentage), as well as the quantified area of blood vessel density, chondrocytes cell roundness, and area of chondrocytes cell percentage, were measured according to the method of Fardin et al. [60] and, Jahejo et al. [12], in addition, the quantification of H&E staining was performed by ImageJ software (ImageJ 1.42q; Pixels 4080 to 3072). The equation “C = 4p (A/P2)” defined circularity; p = ~3.14 mathematical constant, P = perimeter of the cell, and A = area of the cell. The circularity value 1.0 indicates a perfectly circular shape, whereas the elongated polygon is indicated by the value 0.

### Confirmation using quantitative real-time PCR

Besides morphological analysis, it was also necessary to reveal whether the supplementation with Ex-FABP protein in thiram-induced TD chickens also affects angiogenesis and immunity-related genes. Thus, we selected 30 angiogenesis and immunity-related biomarker genes as described by Jahejo et al. [14, 21]. We used Quant StudioTM 6 6's TaKaRa SYBR Premix Ex Taq TM II (RR820A; Takara Bio Inc., Dalian, China). Each real-time PCR reaction included 1 μL of cDNA, 0.15 μL of each reverse and forward primer (10 pmol/μL), 6 μL SYBR Premix Ex Taq II, 0.1 μL of ROX dye II, and 2.6 μL of double H_2_O distillate. The thermal cycles were followed as, 95 °C – 3 min, 42 cycles of 95 °C – 30 s, 55 °C – 30 s, and 72 °C –10 s. Shanghai Generay Biotech Co Ltd synthesized primers, and their specific sequences and accession numbers are outlined in Table 1. The data from quantitative real-time PCR was calculated based on three biological and technical replicates, using the 2-Ct method.

**Table 1:** Details of primers used in this study

Genes	Primers	Accession N0.
*F13*A1	F: GGATGCTTTTGGTCTGATAC	NM_204685.1
	R: CTGCTTGTTGATCTCATTGG	
*CLU*	F: CGTCAGTTCGGTTGGGTCTT	NM_204900.1
	R: CCTCGAGGTTGGGAGTTTTG	
*TBXA2R*	F: GACAGCGAAGTGGAGATGAT	XM_015299775.1
	R: GGAGAGTGGTCTGGATGATG	
*IL1R1*	F: TTTCACGCACCATGAATCTG	NM_205485.1
	R: GAGCCGAGTTCCACTTCAAT	
*RPL*17	F: GTGAACAAGGCTCCCAAAAT	NM_001282277.1
	R: CGGTGAGGATCATCTCAATG	
*ITGB3*	F: TTTGTGGACAAGCCCATTTC	NM_204315.1
	R: CAAACATGGGCAAGCACTTT	
*ITG*AV	F: TCAGTGGTTTGGAGCATCTG	NM_205439.1
	R: AGGCTCTCGCTCTTGTTTTG	
*ITG*B2	F: CAGTGCGTTCAGCAATAAGA	NM_205251.1
	R: AGCACGACGAAACTTCACAT	
*RAC2*	F: GGAGAATACATCCCCACTGT	NM_001201452.1
	R: CCTCTTGTCCAGCAGTATCC	
*ITG*A2	F: TGCTAATAATCCCCGTGTAG	XM_015277561.1
	R: CAGATCTCCTCCCTGTTGAA	
*IQGAP2*	F: CTGGTGAGGCCAGTAAATTG	NM_001277778.1
	R: CGCAAACTCTGAATCGATGA	
*GIT1*	F: CATCACGCTGCAGGAGTACCT	NM_204296.1
	R: CTCATCGCTCAGGCTGTTGT	
*VAV1*	F: CTGGAGACGTTGTAGGGTATG	NT_469922.1
	R: TGGTGAGACGGGTTGGTT	
*ITGA5*	F: CTCCAACTACCCCGAGTACT	KC439457.1
	R: CACCGAATAGCCCATATAAC	
*RAP*1B	F: TCGAGTCAAAGACACTGATG	NM_001007852.1
	R: TAGGTTCTGACCTTGTTCCT	
*FYN*	F: AGGAGTGGTACTTCGGCAAA	NM_205349.2
	R: GTTTCACTTTCCCGGATCAG	
*PTPN11*	F: TGGAGGCAGAAAATCTACTG	NM_204968.1
	R: CGCCTAACAGAGAGAGTGAA	
*PTCH1*	F: TTCCTTCTAGCCCATGCGTTT	NM_204960.2
	R: CTACACTTGCTCCTGTTCGCTTT	
*NCOR2*	F: GGTCCCACACACTTGAC	XM_015275621.1
	R: GGTGGATACTAGGATGGATT	
*MAML3*	F: GCAACCACACGCTGATCATG	XM_015276692.1
	R: CCATCGCAAACTCCATTCTG	
*MHC*II	F: TGCCCGAAACCGACCGTCTG	NM_001318995
	R: TCCAGCACCACCAGCACCTG	
*MyD*88	F: CAGAAAGACCTTCAGTTTGT	NM_001030962
	R: AATGACGACCACCATCCTCC	
*TLR*2	F: ACCTGGCCCATAACAGGATA	AB046119
	R: ATGGAGCTGATTTGGTTGGA	
*TLR3*	F: GCCTAAATATCACGGTACTC	NM_001011691
	R: CACAACAGTGGTAGTGATCA	
*TLR*4	F: GCCCTGAGGGAAACAAACAG	NM_001030693
	R: CACCAAGGTGAATCTTTGCA	
*TLR*5	F: CTGCCAAATCTTCGTGTCTT	FJ915552
	R: ACAGACGGAGTATGGTCAAA	
*TLR*7	F: GGTGTTAGCCACGTGCTTAG	NM_001011688
	R: CCATCCCTGTGCTGATAGAG	
*TLR*15	F: GTGTCCAACTGCTCCATCGT	NM_001037835
	R: GAAGCATGGAAATCCGATTG	
*IL-*7	F: CTTGTTCTGTCGCCAGTGAA	NM_001037833
	R: CTTCGATGTCATGGCTGAGT	
*TRAF*6	F: ATGGAAGCCAAGCCAGAGTT	XM015287208
	R: ACAGCGCACCAGAAGGGTAT	

*F* Forward, *R* Reverse

### Protein-protein interaction (PPI) network construction

In order to find which gene or pair of the gene as protein interacts best within the list of screened immunity and angiogenesis-related genes, the STRING database was used for PPIs following the experiment of Mering et al. [55]. The STRING database was used to find the predicted PPIs interaction based on the detail available in the database. This database working method is on the prediction (neighborhood gene, gene fusion, co-occurrence, experiment co-expression, database, and text mining). We analyzed the immunity and angiogenesis-related genes to find their PPIs, a combined score above > 0.9 was used as the cut-off value. A low confidence PPI (0.150) was used in the STRING database (*Gallus gallus*).

### Statistical analysis

The data was expressed as mean and standard deviation (mean ± SD). A value of *P* < 0.05 was considered statistically significant. Use JMP (version 10) software for statistical analysis.

## Supplementary Information

**Additional file 1.**

**Additional file 2.**

**Additional file 3.**

**Additional file 4.**

**Additional file 5.**

**Additional file 6.**

**Additional file 7.**

**Additional file 8.**

**Additional file 9.**

**Additional file 10.**

**Additional file 11.**

**Additional file 12.**

**Additional file 13.**

**Additional file 14.**

## Data Availability

The datasets generated during the current study are not publicly available on the repository. We have got raw read counts from the sequencing company, which are the same as those generated by HTSeq-counts or feature-counts. The data generated or analyzed during this study are included in this published article and its supplementary information files, Additional files 1, 2, 4, 6, 7, 9, and 13.

## Abbreviations

TD: Tibial dyschondroplasia
*ITG*AV: Integrin alpha-v precursor
CLU: Clusterin precursor
DEGs: Differentially expressed genes
MAPK: Mitogen-activated protein kinase
*TLR*2: Toll-like receptors 2
*TLR*3: Toll-like receptors 3
*TLR*4: Toll-like receptors 4
*TLR5*: Toll-like receptors 5
*TLR*7: Toll-like receptors 7
*TLR*15: Toll-like receptors 15
*IL*-7: Interleukin 7
*TNFR*6: Tumor necrosis factor receptor (TNFR) related factor 6
*MHC*II: Major histocompatibility complex II
*MyD*88: Myeloid differentiation primary response 88
*TBX*A2R: Thromboxane A2 receptor
*IL*1R1: Interleukin-1 receptor type 1 precursor
*RP*L17: Ribosomal protein L17
*ITG*B3: Integrin beta-3 precursor
*ITG*B2: Integrin beta-2 precursor
*RAC*2: Ras-related C3 botulinum toxin substrate 2
*ITG*A2: Integrin alpha-2
*IQGAP*2: IQ motif containing GTPase activating protein 2
*GIT*1: ARF GTPase-activating protein
*VAV*1: Proto-oncogene vav
*ITG*A5: Integrin alpha-IIb-like
*RAP*1B: Ras-related protein Rap-1b precursor
*FYN*: Tyrosine-protein kinase Fyn-like
*PTPN*11: Tyrosine-protein phosphatase non-receptor type 11
*PTCH*1: Protein patched homolog 1
*NCOR*2: Nuclear receptor corepressor 2
*MAML*3: Mastermind-like protein 3
*ATP2*A3: Sarcoplasmic/endoplasmic reticulum calcium ATPase 3
*UBE2*R2: Ubiquitin-conjugating enzyme E2 R2
*CCSAP*: Centriole cilia and spindle-associated protein
*F13*A1: Coagulation factor XIII A chain protein
*SHROOM*2: Shroom2 isoform X6
*RASA3*: Ras GTPase-activating protein 3
Thiram: Tetramethyl thiuram disulfide
GO: Gene ontology
KEGG: Kyoto Encyclopedia of Genes and Genomes
VEGF: Vascular endothelial growth factor signaling pathway
VEGFR2: Vascular endothelial growth factor receptor 2
WDR33: WD Repeat Domain 33
CISH: Cytokine inducible SH2 containing protein
ADA: Adenosine deaminase
BCL2L1: *BCL2* like 1
COMMD7: COMM domain containing 7
ZFYVE27: Zinc finger FYVE-type containing 27
SMARCA1: SWI/SNF related, matrix associated, actin dependent regulator of chromatin, subfamily a, member 1
SLC35B2: Solute carrier family 35 member B2
HSP90AB1: Heat shock protein 90 alpha family class B member 1
GBE: Eye-globin
ERC1: ELKS/RAB6-interacting/CAST family member 1
TAF4B: TATA-box binding protein associated factor 4b
LOC417973: Uncharacterized LOC417973
RUNDC3A: RUN domain containing 3A
KLF4: Kruppel like factor 4
VSIG10L: Uncharacterized protein
ZNF292: Zinc finger protein 292
