# Supplementary material for: Transcriptome-based biomarker gene screening and evaluation of the extracellular fatty acid-binding protein (Ex-FABP) on immune and angiogenesis-related genes in chicken erythrocytes of tibial dyschondroplasia
Source: BMC Genomics. 2022 Apr 22;23:323. doi: 10.1186/s12864-022-08494-9 (PMC9034513; doi:10.1186/s12864-022-08494-9)
Supplement: Supplementary file 3 — Additional file 3. [file 12864_2022_8494_MOESM3_ESM.docx]

Additional file 3: Supplementary Fig. 1 Volcano Plot of DEGs showing the gene expression on 6^th^ day vs 2^nd^ day (6d vs 2d), 15^th^ day vs 2^nd^ day (15d vs 2d), 15d vs 6^th^ day (15d vs 6d). The red color represents up-regulated gene expression, and the black dots represent non-significant genes. The vertical axis represents log^10,^ and the horizontal axis refers to log^2^ fold change.

**
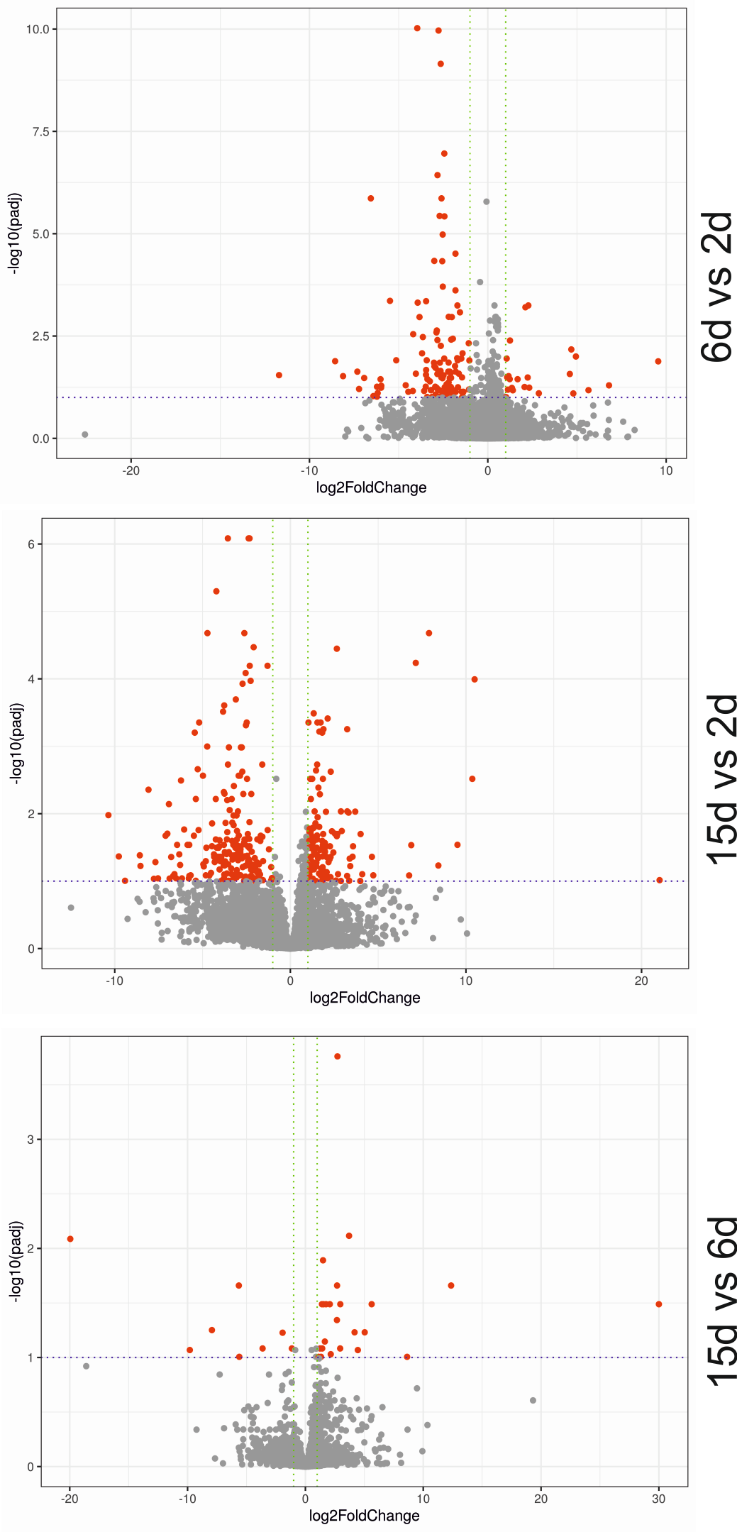
**
