# Supplementary material for: Transcriptome-based biomarker gene screening and evaluation of the extracellular fatty acid-binding protein (Ex-FABP) on immune and angiogenesis-related genes in chicken erythrocytes of tibial dyschondroplasia
Source: BMC Genomics. 2022 Apr 22;23:323. doi: 10.1186/s12864-022-08494-9 (PMC9034513; doi:10.1186/s12864-022-08494-9)

Additional file 8: Supplementary Fig. 3 Gene ontology (GO) annotation of differentially expressed genes. The GO annotation plot of DEGs showing the differentially expressed gene expression on the 6th day vs 2nd day (6d vs 2d), 15th day vs 2nd day (15d vs 2d), 15d vs 6th day (15d vs 6d).

A-Up-regulated and down-regulated DEGs on 15th day vs 2nd day. B- Up-regulated and down-regulated DEGs on 15th day vs 6th day. C- Up-regulated and down-regulated DEGs on 6th day vs 2nd day. The vertical presentation shows the different categories, and the horizontal presentation showing the number of annotated genes. The plot was ranked with *P-*value.


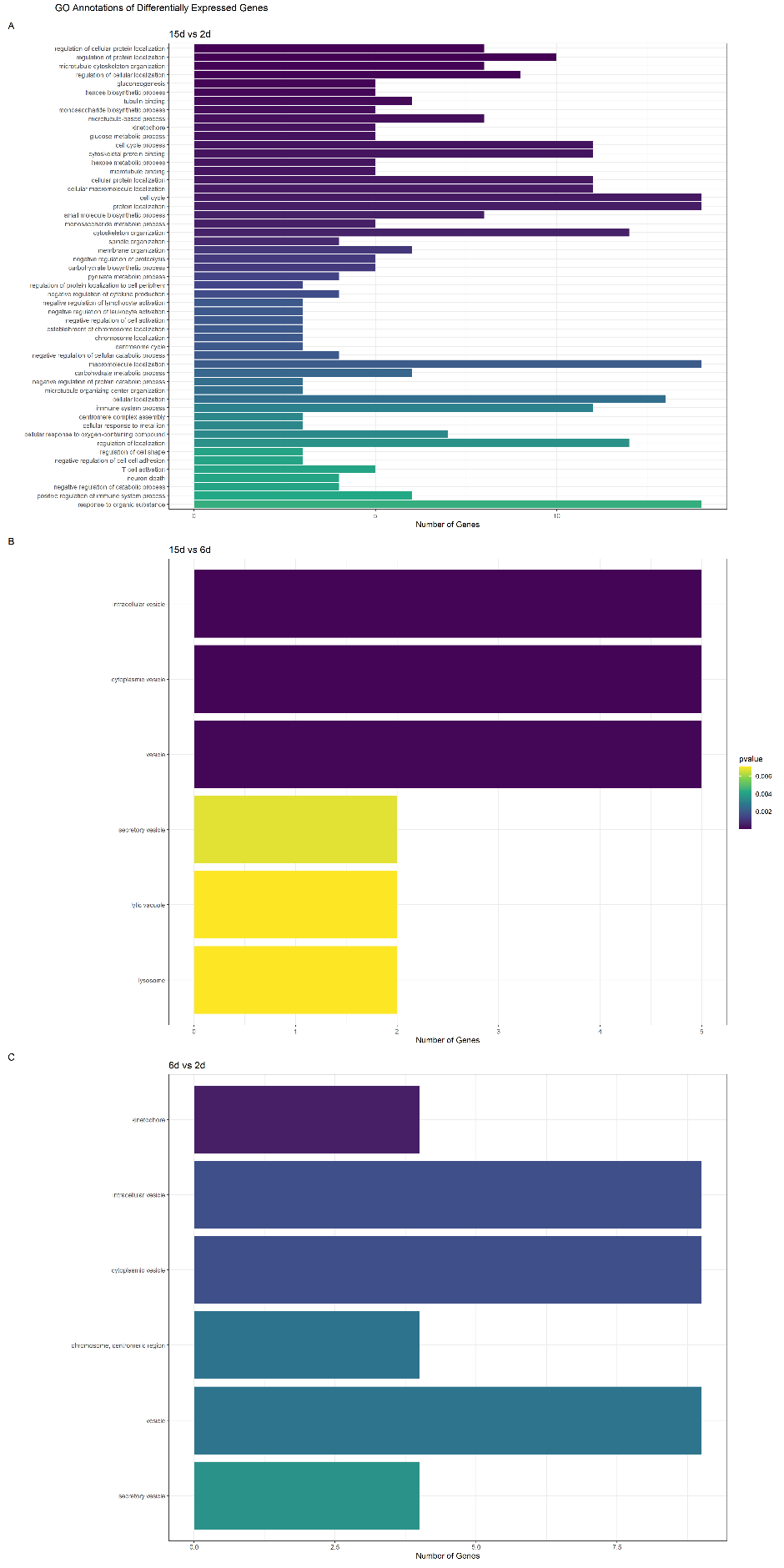

Supplement: Supplementary file 8 — Additional file 8. [file 12864_2022_8494_MOESM8_ESM.docx]
