# Supplementary material for: Transcriptome-based biomarker gene screening and evaluation of the extracellular fatty acid-binding protein (Ex-FABP) on immune and angiogenesis-related genes in chicken erythrocytes of tibial dyschondroplasia
Source: BMC Genomics. 2022 Apr 22;23:323. doi: 10.1186/s12864-022-08494-9 (PMC9034513; doi:10.1186/s12864-022-08494-9)

Additional file 11: Supplementary Fig. 5 Assessment of broiler chicken equilibrium condition and tibia bone morphometry on 6 and 15 days.

EC - equilibrium position (body steadiness on the gravity center); r -radius of the sphere; h - internal shaft. Tibia to toe length, tibial dyschondroplasia occurrence score (TD score), the width of the growth plate (GP width), midline-diameter of the tibia bone (T. Mid-diameter), and length of the tibia was recorded.


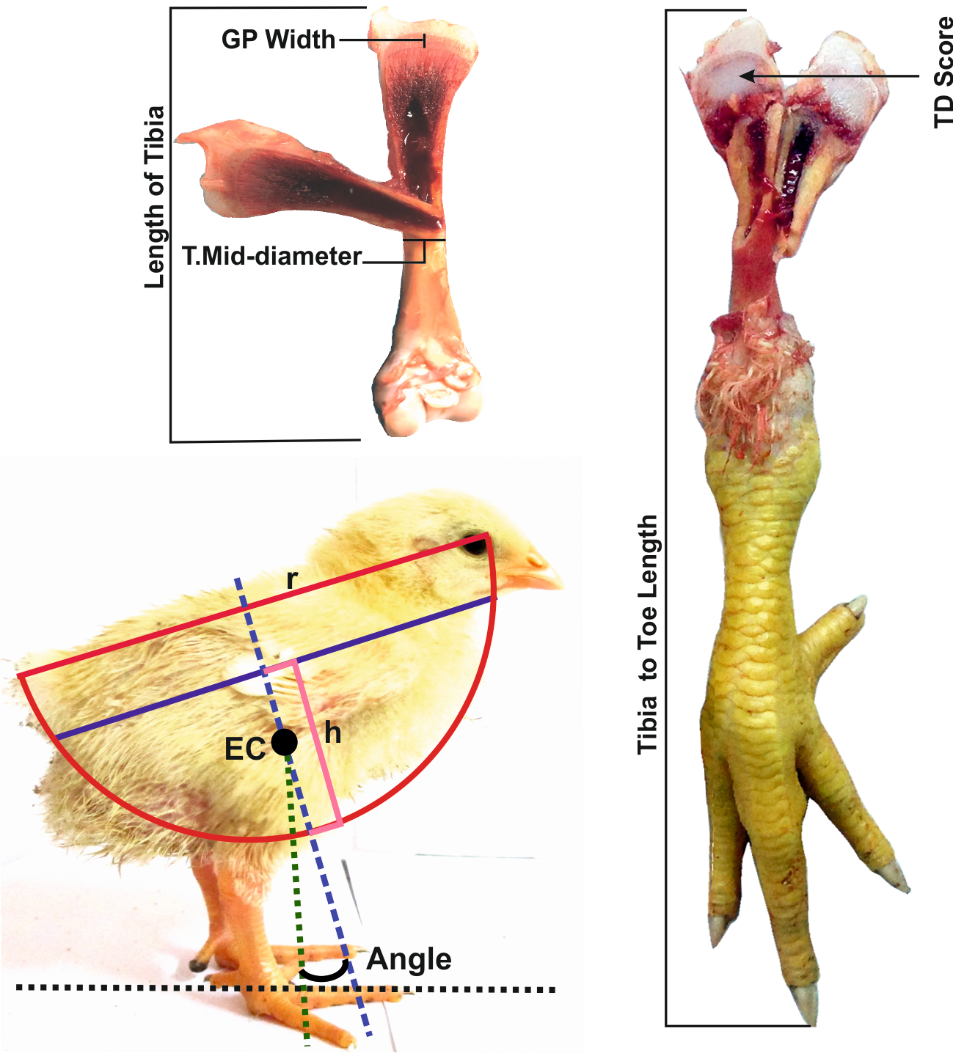

Supplement: Supplementary file 11 — Additional file 11. [file 12864_2022_8494_MOESM11_ESM.docx]
