# Supplementary material for: Transcriptome-based biomarker gene screening and evaluation of the extracellular fatty acid-binding protein (Ex-FABP) on immune and angiogenesis-related genes in chicken erythrocytes of tibial dyschondroplasia
Source: BMC Genomics. 2022 Apr 22;23:323. doi: 10.1186/s12864-022-08494-9 (PMC9034513; doi:10.1186/s12864-022-08494-9)

Additional file 12: Supplementary Fig. 6 Original, unprocessed, and as full as possible length version of gel electrophoresis images.


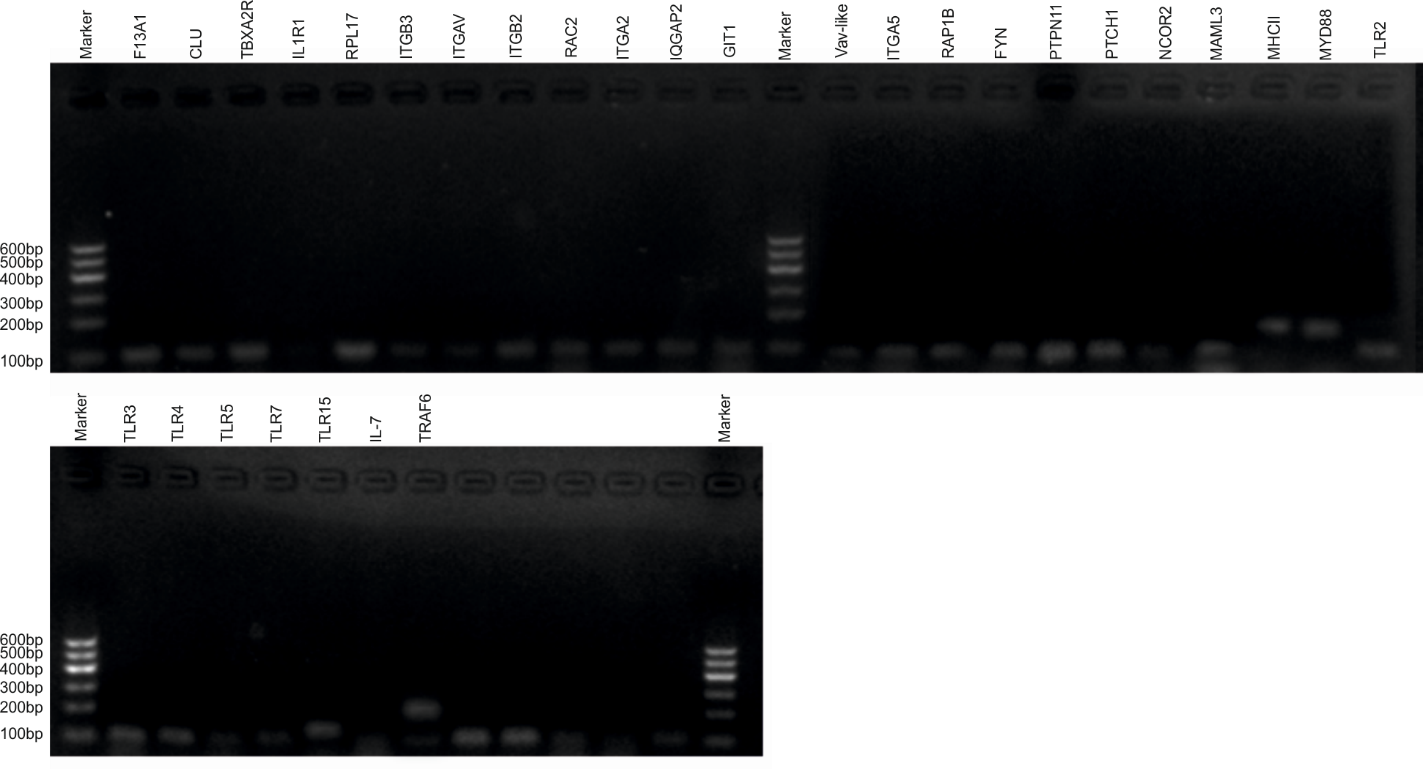

Supplement: Supplementary file 12 — Additional file 12. [file 12864_2022_8494_MOESM12_ESM.docx]
