# Supplementary material for: Transcriptome-based biomarker gene screening and evaluation of the extracellular fatty acid-binding protein (Ex-FABP) on immune and angiogenesis-related genes in chicken erythrocytes of tibial dyschondroplasia
Source: BMC Genomics. 2022 Apr 22;23:323. doi: 10.1186/s12864-022-08494-9 (PMC9034513; doi:10.1186/s12864-022-08494-9)

Additional file 14: Supplementary Fig. 7 Protein‑protein interaction network of angiogenesis and immune-related genes using STRING (<https://string-db.org/>). Proteins are presented in the way of gene symbols. The implications of different colorful lines between the proteins are presented below the network map.
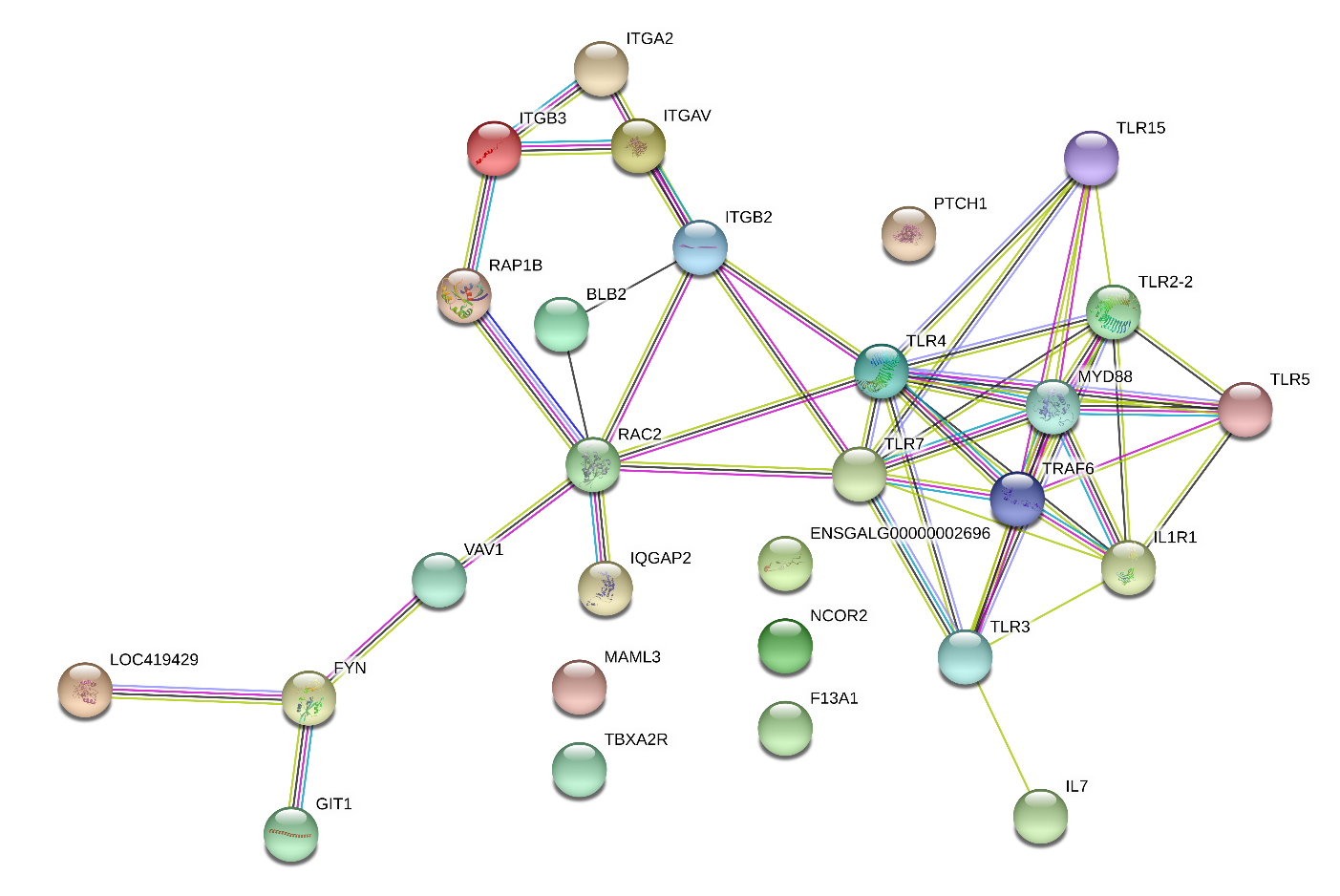

Supplement: Supplementary file 14 — Additional file 14. [file 12864_2022_8494_MOESM14_ESM.docx]
